# The application of a clinical-multimodal ultrasound radiomics model for predicting cervical lymph node metastasis of thyroid papillary carcinoma

**DOI:** 10.3389/fonc.2024.1507953

**Published:** 2025-01-17

**Authors:** Chang Liu, Shangjie Yang, Tian Xue, Qian Zhang, Yanjing Zhang, Yufang Zhao, Guolin Yin, Xiaohui Yan, Ping Liang, Liping Liu

**Affiliations:** ^1^ Department of Interventional Ultrasound, First Hospital of Shanxi Medical University, Taiyuan, China; ^2^ Department of Ultrasound, Xi'an Central Hospital, Xi'an, China; ^3^ Department of Medical Imaging, Shanxi Medical University, Taiyuan, China; ^4^ Department of Ultrasound, Shanxi Maternal and Child Health Care Hospital, Shanxi Children's Hospital, Taiyuan, China; ^5^ Department of Interventional Ultrasound, Fifth Medical Center, Chinese People's Liberation Army General Hospital, Beijing, China

**Keywords:** papillary thyroid carcinoma (PTC), cervical lymph node metastasis, radiomic, multimodal ultrasound imaging, contrast-enhanced ultrasound (CEUS), strain elastography-ultrasound (SE-US)

## Abstract

**Background:**

PTC (papillary thyroid cancer) is a lymphotropic malignancy associated with cervical lymph node metastasis (CLNM, including central and lateral LNM), which compromises the effect of treatment and prognosis of patients. Accurate preoperative identification will provide valuable reference information for the formulation of diagnostic and treatment strategies. The aim of this study was to develop and validate a clinical-multimodal ultrasound radiomics model for predicting CLNM of PTC.

**Methods:**

One hundred sixty-four patients with PTC who underwent treatment at our hospital between March 2016 and December 2021 were included in this study. The patients were grouped into a training cohort (n=115) and a validation cohort (n=49). Radiomic features were extracted from the conventional ultrasound (US), contrast-enhanced ultrasound (CEUS) and strain elastography-ultrasound (SE-US) images of patients with PTC. Multivariate logistic regression analysis was used to identify the independent risk factors. FAE software was used for radiomic feature extraction and the construction of different prediction models. The diagnostic performance of each model was evaluated and compared in terms of the area under the curve (AUC), sensitivity, specificity, accuracy, negative predictive value (NPV) and positive predictive value (PPV). RStudio software was used to develop the decision curve and assess the clinical value of the prediction model.

**Results:**

The clinical-multimodal ultrasound radiomics model developed in this study can successfully detect CLNM in PTC patients. A total of 3720 radiomic features (930 features per modality) were extracted from the ROIs of the multimodal images, and 15 representative features were ultimately screened. The combined model showed the best prediction performance in both the training and validation cohorts, with AUCs of 0.957 (95% CI: 0.918–0.987) and 0.932 (95% CI: 0.822–0.984), respectively. Decision curve analysis revealed that the combined model was superior to the other models.

**Conclusion:**

The clinical-multimodal ultrasound radiomics model constructed with multimodal ultrasound radiomic features and clinical risk factors has favorable potential and high diagnostic value for predicting CLNM in PTC patients.

## 1 Introduction

During the past several decades, an increasing number of thyroid nodules have been detected worldwide. PTC, the most common histological type of thyroid malignancy (1), is an indolent carcinoma characterized by slow progression. Although the mortality rate associated with PTC remains low, CLNM usually occurs in 30% to 80% of PTC patients without obvious early manifestations (2), thus increasing the difficulty of surgery and the risk of recurrence (3, 4). For these reasons, some experts recommend central lymph node dissection (CLND) during surgery to improve the therapeutic effect (5). However, it is still unclear whether prophylactic CLND is beneficial for PTC patients (6). Therefore, early and precise identification of metastatic lymph nodes can effectively guide clinicians in dissecting appropriate lymph nodes.

As the preferred method for examining thyroid nodules, ultrasound is uniquely advantageous in that it is repeatable, safe and noninvasive, allows real-time dynamic exploration, and patients are not exposed to radiation. It is well known that the characteristics of primary tumors are particularly correlated with invasiveness and metastasis. As a result, we can assess the status of the LN by analyzing the grayscale, features on CEUS images and characteristics of PTC on SE-US images (7, 8). Studies have shown that preoperative ultrasound is beneficial for the diagnosis of lymph node metastasis in PTC patients to some extent. However, ultrasound diagnosis heavily depends on the experience and subjective judgment of the operators. The sensitivity of ultrasound in the detection of CLNM is low, and 20–80% of PTC patients with clinically negative lymph nodes have microscopic lymph node metastasis (9, 10). The low rate of detection of such lymph nodes has forced patients with suspected thyroid tumors to undergo fine-needle aspiration biopsy (FNAB) and prophylactic lymph node dissection, both of which are invasive and redundant for most patients without lymph node metastasis. Thus, a more impersonal but precise method is needed for the preoperative localization of PTC in patients with a high risk of CLNM.

Dutch Professor Lambin et al. (11) proposed the concept of radiomics for the first time in 2012. In domestic and international studies, researchers have applied radiomics technology in the examination of ultrasound images to distinguish malignant thyroid nodules from benign nodules and predict lymph node metastasis (12–14). However, few studies involve the application of radiomics in the examination of CEUS images to predict lymph node metastasis. Therefore, in this study, radiomics was used to extract features from the US, CEUS and SE-US images of patients with PTC. Thus, we constructed three models (clinical, radiomic, and clinical-radiomic models) to explore the accuracy of predicting lymph node metastasis using the images of different ultrasound techniques as well clinical features. Decision curve analysis (DCA) was performed to evaluate the clinical value of each prediction model. It can better predict CLNM in patients with PTC before surgery and provide more valuable reference information for clinical therapy and observation.

## 2 Materials and methods

### 2.1 Patients

One hundred sixty-four patients with PTC confirmed via pathological examination of samples retrieved during surgery at our hospital between March 2016 and December 2021 were enrolled in this study. The patients were randomly classified into a training cohort (n=115) and a validation cohort (n=49) at a ratio of 7:3. Moreover, the included patients were divided into a lymph node metastasis group and a non-metastasis group.

The inclusion criteria were as follows: ①nodules confirmed as PTC via pathological examination; ②preoperative US, CEUS and SE-US examinations, and clear and complete images in DICOM format; ③no preoperative radiotherapy, chemotherapy or other related anticancer treatment; and ④signed informed consent form prior to CEUS examination.

The exclusion criteria were as follows: ①nodules unable to be confirmed via pathological examination; ②unclear or incomplete images due to the presence of gas, a complex anatomical structure, or other factors; ③pregnancy or lactation; and ④history of severe allergies or serious cardiopulmonary disease.

### 2.2 Multimodal ultrasound image acquisition

All ultrasound examinations were performed with a GE LOGIQ E9 color Doppler ultrasound diagnostic instrument equipped with an ML6-15 (6–15 MHz) linear array transducer for US and SE-US and a 9 L (2–9 MHz) transducer for CEUS. The patient was placed in the supine position with full exposure of the anterior and lateral sides of the neck. US, SE-US and CEUS were performed successively, and clear images were recorded, with each image containing as many typical malignant features as possible.

The contrast agent used in this study was sulfur hexafluoride microbubbles (SonoVue^®^, Bracco, Milan, Italy). The patients were given 1.5ml of contrast agent as a bolus through the antecubital vein, followed by 5ml normal saline immediately, and the timing function was turned on simultaneously. About 90s of the dynamic contrast images were stored, and the frame with the best enhancement degree between the early and late enhancement was selected for storage.

Confirm that the target nodule is in the appropriate position of the image interface, then activate the elastography mode, adjust the sampling frame until it is twice as much as the nodule size and include the surrounding normal glandular tissue for comparison. Ask the patient to hold his breath, place the probe vertically on the body surface and make small vibration with relatively uniform force, observe the color image of elastic imaging. When the color in the sampling frame is no mosaic and the pressure indicator on the display screen shows full green, fix the frame and access the image after 3-5s stability.

### 2.3 Analysis of ultrasound images and basic clinical features

The general clinical information included sex and age (≤45 years, >45 years). On the basis of the stored multimodal ultrasound images, two experienced physicians analyzed and recorded the relevant characteristics of the thyroid nodules, including nodule size, echo, shape, margin, calcification, A/T, multifocality, contrast-enhanced ultrasound pattern, and capsular contact range (they were divided into four groups: 0, <25%, 25%–50%, and >50% according to the proportion of the contact area accounting for the whole circumference of the cancer nodule).

### 2.4 Region of interest segmentation and feature extraction

First, representative ultrasound images were selected from the image set, and the best original image in DICOM format was exported as the source data. Two experienced technicians (doctor A for 8 years and doctor B for 10 years) used ITK-SNAP, which is open-source software, to manually outline the ROI along the nodule boundary. The double-blind method was employed for the segmentation of nodules, and the repeatability and stability of the segmented images were evaluated using intraclass and interclass correlation coefficients (ICCs). The ultrasound images of 30 patients were randomly selected by two doctors for segmentation; these images were resegmented two weeks later by Doctor A using the same procedure, and image feature extraction was considered reproducible if the ICC was >0.75. Radiomics features, were extracted from the original image, wavelet transform and other transformed derived images using FAE Pro 0.3.6. These features include shape features, first-order histogram features, and second-order histogram features. Shape features provide information about the size and form of the tumor, which is helpful for evaluating the growth pattern and invasiveness of the tumor. First-order histogram features reflect the fundamental properties of the image, such as the symmetry, uniformity, and local intensity variations of the gray level distribution. These features are useful for describing the heterogeneity and internal structure of the tumor. Second-order histogram features, also known as texture features, encompass Gray Level Co-occurrence Matrix (GLCM), Gray Level Size Zone Matrix (GLSZM), Neighbouring Gray Tone Difference Matrix (NGTDM), Gray Level Run-Length Matrix (GLRLM), and Gray Level Dependence Matrix (GLDM). These features capture the complex texture information within the image, aiding in the description of the microstructure and heterogeneity of the tumor.

### 2.5 Feature selection and radiomics model establishment

The risk factors identified from the basic clinical and ultrasound features of PTC patients (nodule size, echo, microcalcification, multifocality, capsule contact range and enhancement pattern) were combined with the extracted ultrasound radiomic features to form a new feature set, which was normalized by Z score normalization. The Pearson correlation coefficient (PCC) was used to reduce the dimensionality of the feature matrix high-dimensional space, and the REF was used to select the key features before building the model, which aims to select the feature-based classifier via recursive elimination while considering a smaller set of features. Logistic regression with the LASSO constraint was selected as the classifier to construct the clinical-multimodal ultrasound radiomics model.

**Figure 1:**
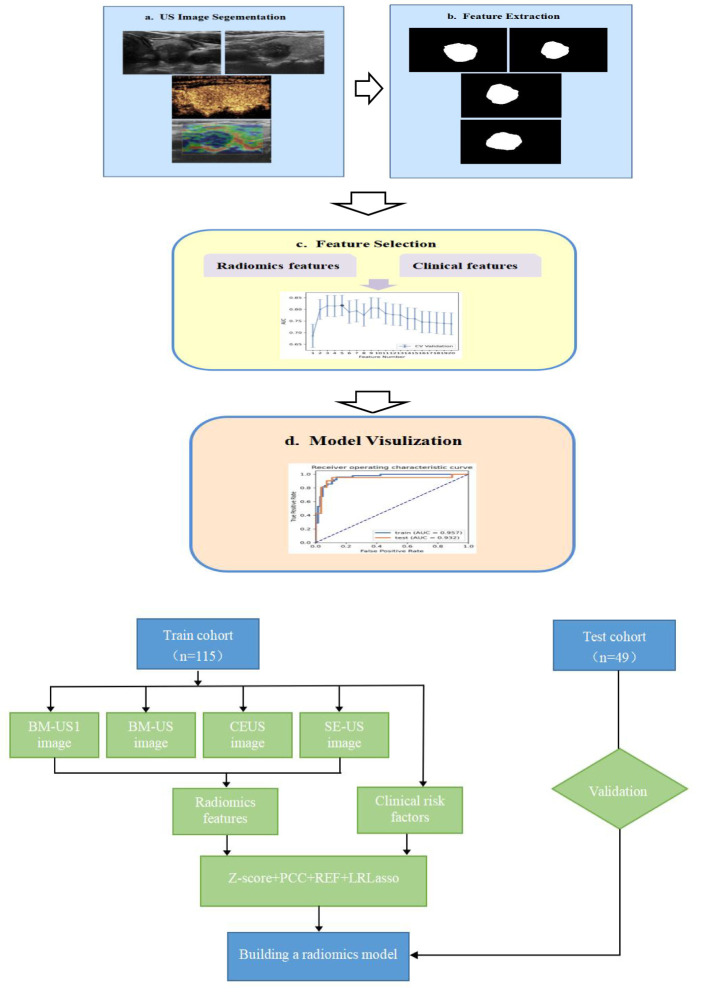
Modeling flow chart **(A)** and radomics workflow **(B)** of the combined model for predicting CLNM in PTC patients. (BM-US1 represents the conventional two-dimensional ultrasonic cross-section, BM-US represents the conventional two-dimensional ultrasonic longitudinal section).

We extracted 3720 quantitative features (930 features per modality) from the ROIs of the US, CEUS and SE-US images of each patient. The feature datasets were established with basic clinical or ultrasound features, and each feature was normalized. Pearson’s correlation coefficient (PCC) or principal component analysis (PCA) was used to reduce the row-space dimension of the feature matrix, making each feature relatively independent. Representative features were selected using ANOVA, REF, Relief, and KW methods. The selected critical feature set was subjected to six classifiers, including support vector machine (SVM), linear discriminant analysis (LDA), logistic regression (LR), least absolute shrinkage and selection operator (LASSO), decision tree (DT), and naive Bayes (NB), to construct the prediction model. To avoid overfitting and underfitting, 10-fold cross validation was performed to test the prediction models. The process of constructing the models is shown in **Figure 1**.

### 2.6 Comparison and evaluation of prediction models

Three prediction models for CLNM in PTC patients were developed in this study: a clinical model, a multimodal ultrasound radiomics model, and a combination of a clinical-multimodal ultrasound radiomics model. Finally, the AUC value, sensitivity, specificity, accuracy, PPV and NPV of different prediction models were compared to evaluate their predictive efficacy, and the best model for predicting CLNM in PTC patients was selected. To further evaluate the clinical application value of the prediction model, we quantified the net benefit of the joint prediction model at different threshold probabilities and plotted the clinical decision curve.

### 2.7 Statistical analysis

All the statistical analyses were performed using SPSS 22.0. The measurement data were analyzed by two independent sample t tests or Mann−Whitney U nonparametric tests and are presented as means ± standard deviations. The χ2 test or Fisher’s exact test was used for comparisons between groups. Independent risk factors were identified via multivariate logistic regression analysis to develop a clinical prediction model. FAE software was used for radiomics feature extraction and prediction model construction. Intraclass and interclass correlation coefficients were employed to assess the reproducibility of radiomics feature extraction in this study. The ROC curve of each prediction model was drawn, and the AUC value, sensitivity, specificity, accuracy, NPV and PPV were calculated. The Delong test was performed with MedCalc 19.3.1 software to compare the ROC curves of multiple models. The R Studio software package “rmda” was used to analyze the decision curve to further evaluate the clinical utility of the prediction model. A P value < 0.05 indicated statistical significance.

## 3 Results

### 3.1 Clinical and ultrasound basic features of PTC

A total of 164 patients were enrolled in this study (115 in the training cohort and 49 in the validation cohort), including 27 males and 137 females aged 24-79 years. The average age of the patients was 45.18 ± 10.29 years. There were 70 patients in the CLNM group and 94 patients in the non-CLNM group. **Table 1** shows that there were significant differences in nodule size (P<0.001), echo (P=0.046), microcalcification (P=0.035), multifocality (P<0.001), capsular contact range (P<0.001) and enhancement pattern (P=0.038) between the CLNM group and the non-CLNM group in the training cohort. In the validation cohort, nodule size (P=0.017), microcalcification (P=0.006), multifocality (P=0.021), and range of capsular contact (P=0.003) were significantly different.

**Table 1:** Clinical and ultrasound basic features of PTC.

	Training cohort(n=115)			Validation cohort(n=49)		
	CLNM(+)(n=49)	CLNM(-)(n=66)	*P*	CLNM(+)(n=21)	CLNM(-)(n=28)	*P*
**Age** ( $\bar{\text{X}}$ ±s)	44.80±10.46	46.14±9.01		44.71±12.44	43.96±10.84	
≤45	25(51.02)	30(45.45)	0.555	10(47.62)	16(57.14)	0.509
>45	24(48.98)	36(54.55)		11(52.38)	12(42.86)	
**Gender**						
male	7(14.29)	11(16.67)	0.728	6(28.57)	3(10.71)	0.110
Female	42(85.71)	55(83.33)		15(71.43)	25(89.29)	
**Nodule size**(cm)						
<1	14(28.57)	51(77.27)	<0.001	7(33.33)	19(67.85)	0.017
≥1	35(71.43)	15(22.73)		14(66.67)	9(32.14)	
**Echogenicity**						
hypoechoic	38(77.55)	60(90.91)	0.046	18(85.71)	27(96.43)	0.175
iso/hyperechoic	11(22.45)	6(9.09)		3(14.29)	1(3.57)	
**Shape**						
regular	8(16.33)	13(19.70)	0.644	1(4.76)	5(17.86)	0.219
Irregular	41(83.67)	53(80.30)		20(95.24)	23(82.14)	
**Margin**						
well-defined	18(36.73)	24(36.36)	0.967	8(38.10)	10(35.71)	0.864
ill-defined	31(63.27)	42(63.64)		13(61.90)	18(64.29)	
**Microcalcification**						
yes	40(81.63)	42(63.64)	0.035	19(90.48)	15(53.57)	0.006
no	9(18.37)	24(36.36)		2(9.52)	13(46.43)	
**A/T (aspect ratio)**						
>1	19(38.78)	31(46.97)	0.381	6(28.57)	15(53.57)	0.080
≤1	30(61.22)	35(53.03)		15(71.43)	13(46.43)	
**Multifocality**						
Yes	24(48.98)	8(12.12)	<0.001	8(38.10)	2(7.14)	0.021
No	25(51.02)	58(87.88)		13(61.90)	26(92.86)	
**Capsular contact**	**range**					
0	3(6.12)	16(24.24)	<0.001	2(9.52)	6(21.43)	0.003
<25%	4(8.16)	21(31.82)		1(4.76)	9(32.14)	
25%-50%	16(32.65)	23(34.85)		4(19.05)	6(21.43)	
>50%	26(53.06)	6(9.09)		14(66.67)	7(25.00)	
**CEUS pattern**						
low	32(65.31)	48(72.73)	0.038	14(66.67)	22(78.57)	0.745
equal	9(18.37)	16(24.24)		6(28.57)	5(17.86)	
high	8(16.33)	2(3.03)		1(4.76)	1(3.57)	

### 3.2 Clinical prediction model construction

For the basic clinical and ultrasonic characteristics, the variables that were significantly different between the CLNM group and the non-CLNM group in the univariate analysis of the training cohort were incorporated into the multivariate logistic regression analysis. After variable screening, a nodule size ≥1 cm (P=0.041), multifocality (P<0.001), and a contact range with a capsule >50% (P=0.021) were statistically significant (**Table 2**). These three variables were used as independent risk factors for predicting cervical CLNM in PTC patients to construct a clinical prediction model. The AUC of the model in the training cohort was 0.841 (95% CI95% CI: 0.764–0.918), and the sensitivity, specificity, and accuracy were 67.3%, 90.9% and 80.9%, respectively. In the validation cohort, the AUC was 0.777 (95% CI: 0.636–0.884), and the sensitivity, specificity, and accuracy were 76.2%, 67.9%, and 71.4%, respectively.

**Table 2:** Multivariate Logistic regression analysis of cervical lymph node metastasis in PTC.

variables	*β*	*S. E*	Wald	*P*	*OR*	95% CI for OR	
						Lower Limit	Upper Limit
**Nodule size**	1.169	0.572	4.183	0.041	3.219	1.050	9.867
**Multifocality**	1.983	0.570	12.124	<0.001	7.266	2.379	22.190
**Capsular contact range>50%**	2.069	0.894	5.353	0.021	7.918	1.372	8.816
**Constant**	-2.162	0.682	10.054	0.002	0.115		

### 3.3 Multimodal ultrasound radiomics model construction

**Figure 2:**
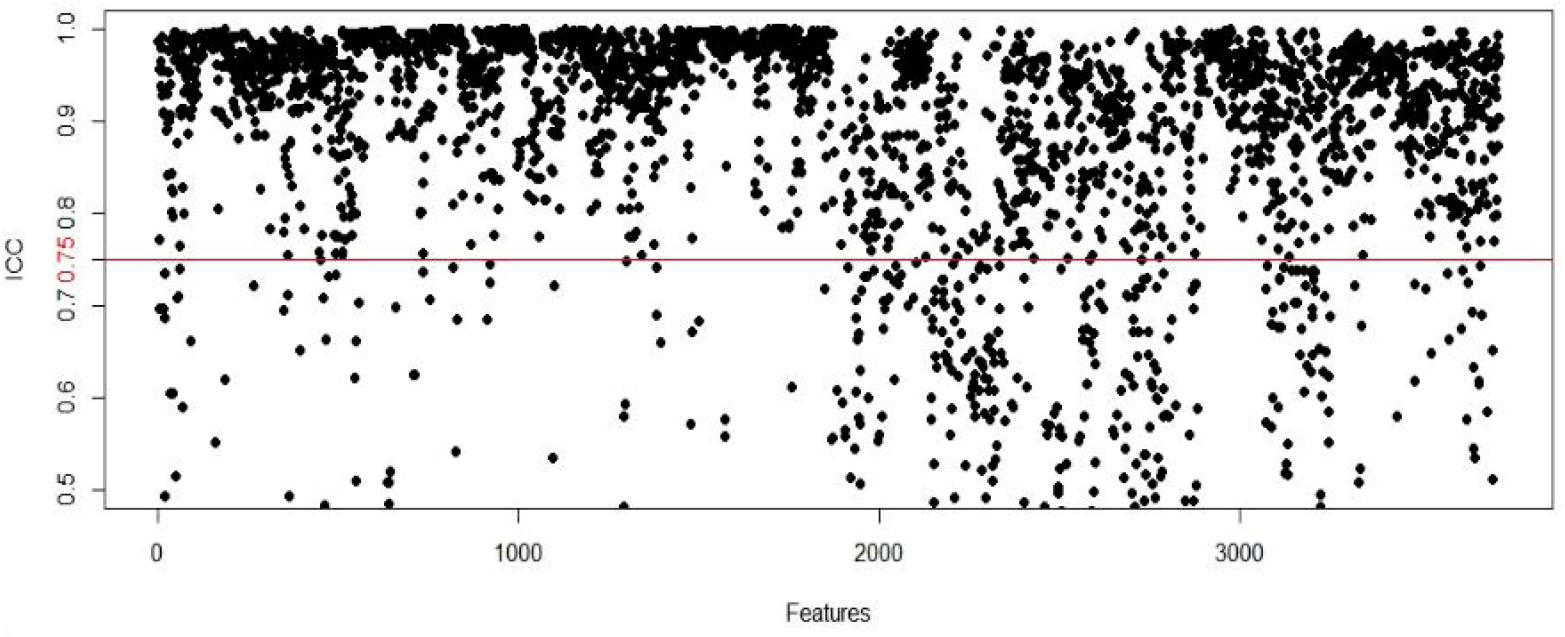
The ICC between docter A and docter B.

**Figure 3:**
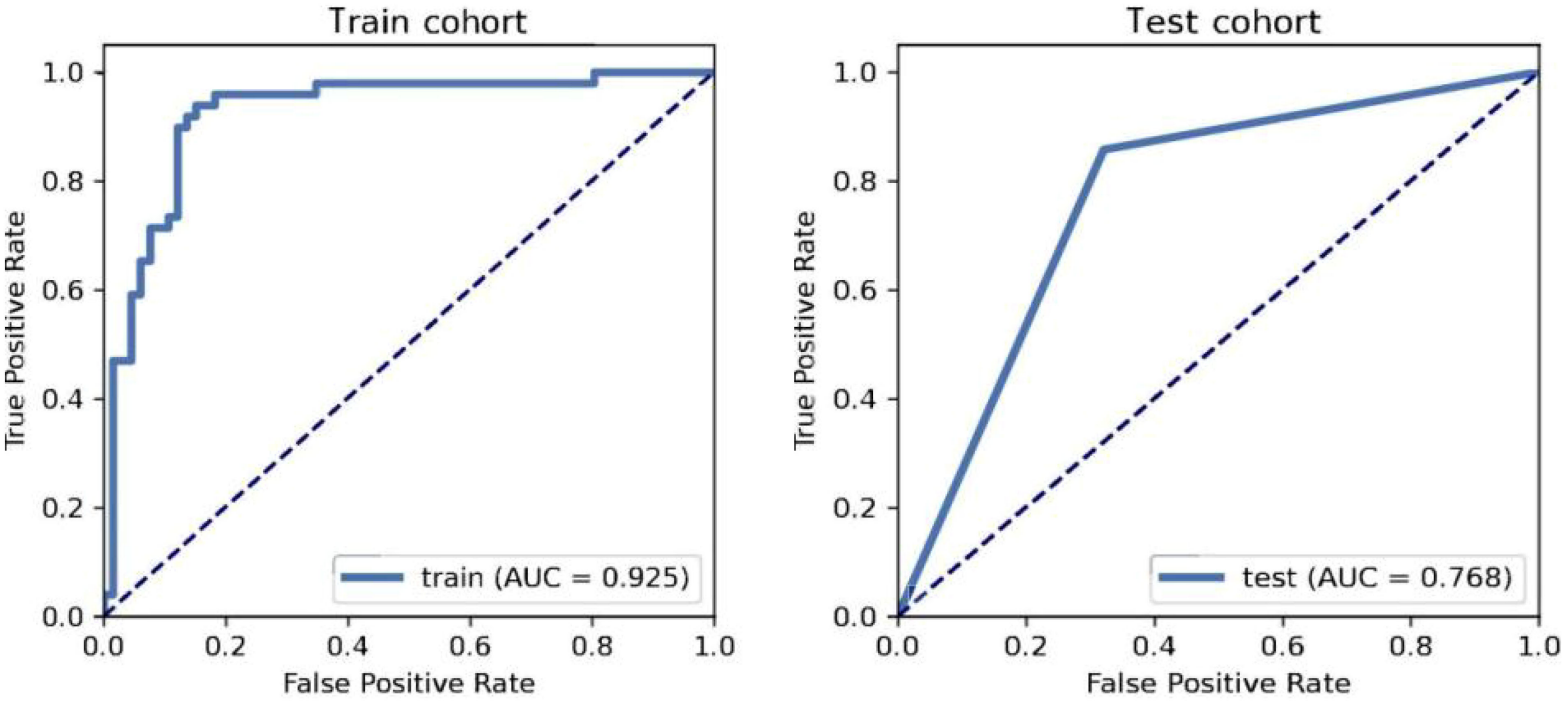
ROC curve performance of multimodal ultrasound radiomics model for PTC patients.

In this study, a total of 3720 features (930 features per modality) were extracted from the ROIs on the US, CEUS and SE-US images. The interobserver agreement test revealed that 3665 intragroup ICCs were greater than 0.75 and that 3273 intergroup ICCs were greater than 0.75, indicating that the repeatability and reliability of image feature extraction were good (**Figure 2**). After 502 radiomic features with ICCs < 0.75 were excluded, the remaining radiomic features were subjected to mean normalization to avoid overfitting of the model, feature dimensionality reduction using PCA and feature selection using recursive feature elimination (REF), 15 representative features were ultimately screened via 10-fold cross-validation, and an LDA classifier was subsequently selected to construct the best radiomics model. The AUC of the model in the training cohort was 0.925 (95% CI: 0.865-0.973); the sensitivity, specificity and accuracy were 93.4%, 84.9% and 88.7%, respectively; the AUC in the validation cohort was 0.768 (95% CI: 0.625-0.876); and the sensitivity, specificity and accuracy were 85.7%, 67.9% and 75.5%, respectively (**Figure 3**).

### 3.4 Clinical-multimodal ultrasound radiomics model construction

**Figure 4:**
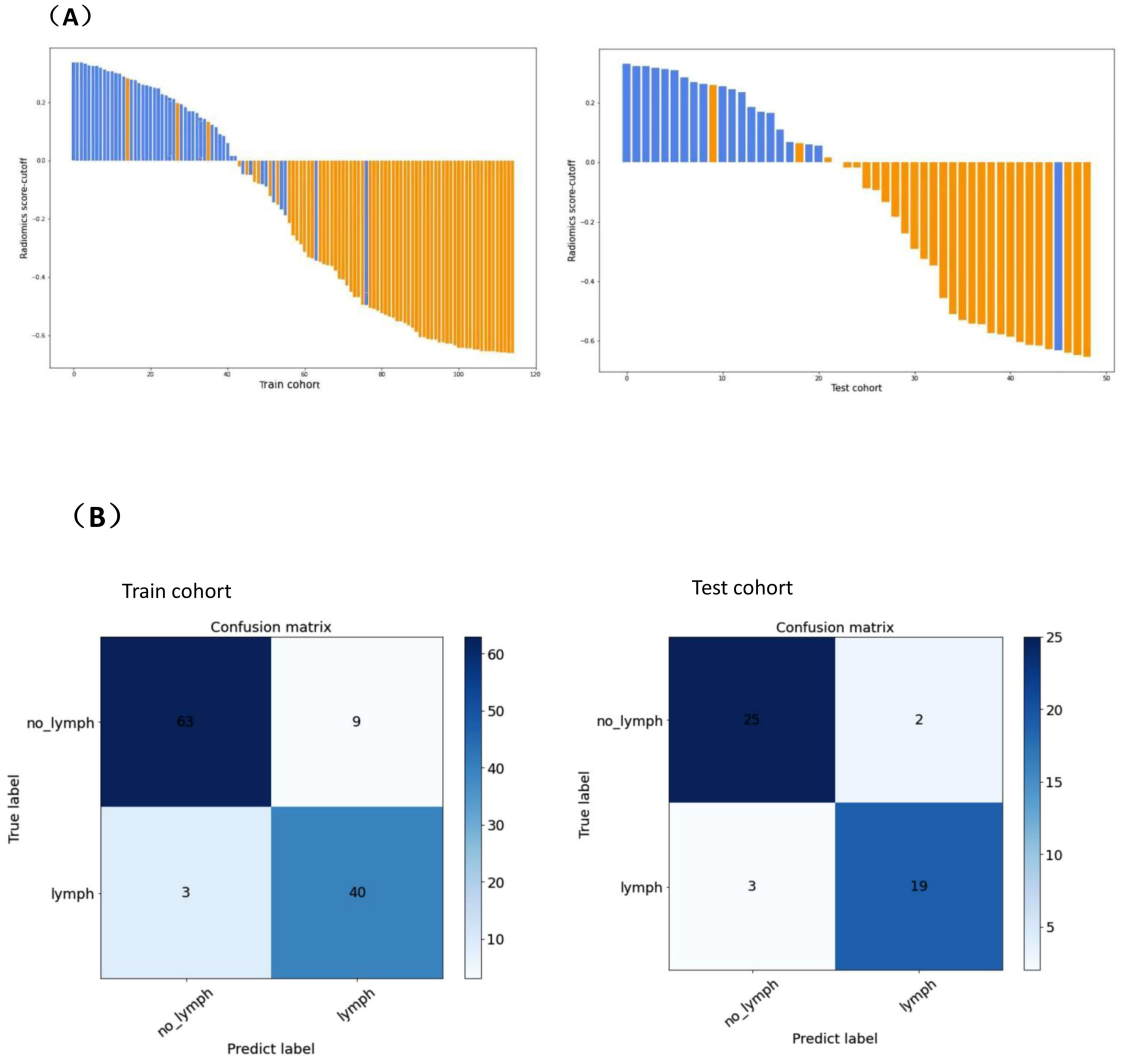
Predicted value distribution (A) and optimal confusion matrix (B) of combined prediction model.(A)The X-axis on the horizontal axis represents the number of PTC patients, and the Y-axis on the vertical axis represents the difference between the predictive value and the cut-off value of radiomics. A positive difference means the model predicts positive lymph node metastasis, and a negative difference means the model predicts negative lymph node metastasis. Blue represents the actual pathology of lymph nodes with metastasis, orange represents the actual pathology of lymph nodes without metastasis. (B) denotes the number of observations in the misclassification class and the alignment class predicted by the combined model for PTC CLNM in the training cohort and validation cohort. “lymph” represents lymph node metastasis, “no_lymph” represents without lymph node metastasis , “Predict label” represents model prediction index, and “True label” represents actual pathological index.

To determine the hyperparameters of the clinical-multimodal ultrasound model, such as the number of features, we applied 10-fold cross-validation to the training dataset and set the hyperparameters according to the performance of the model on the validation dataset. The final results revealed that the model based on 20 key features (3 clinical ultrasound features and 17 radiomics features) achieved the highest AUC in the validation dataset. The AUC, sensitivity, specificity and accuracy of the combined prediction model in the training cohort and validation cohort were 0.957 (95% CI: 0.918-0.987), 95.9%, 86.4%, 90.4% and 0.932 (95% CI: 0.822-0.984), 95.2%, 89.3%, and 91.8%, respectively. The distribution plots of the radiomics predictive value and the best confusion matrix of the clinical-multimodal ultrasound radiomics model are shown in **Figure 4**, which more intuitively shows the consistency between the radiomics combined model and the actual pathological conditions in predicting CLNM of PTC, and the model shows good predictive efficiency.

### 3.5 Diagnostic performance comparison of different models

**Figure 5:**
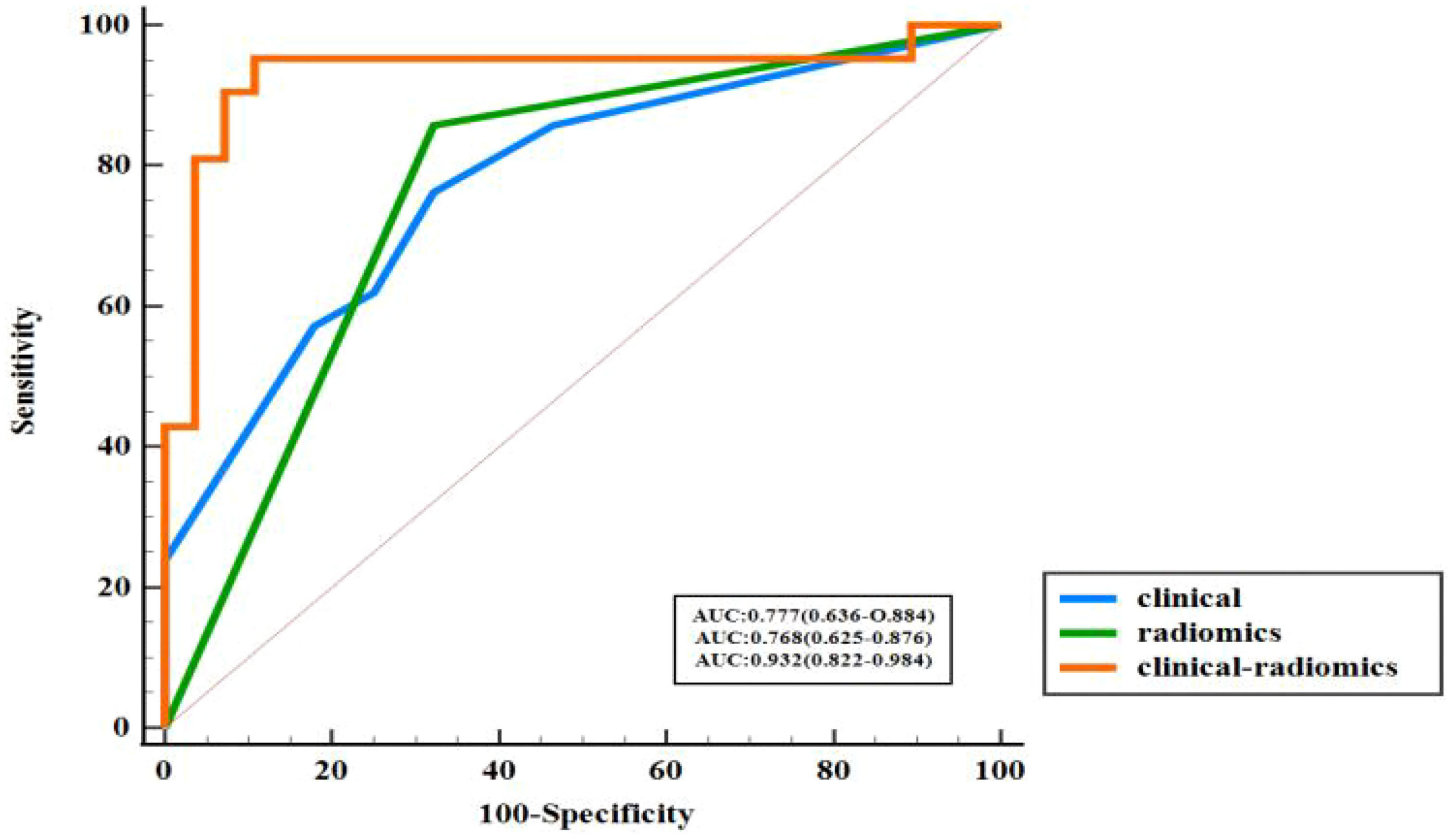
ROC curve of three models in validation cohort.

**Figure 6:**
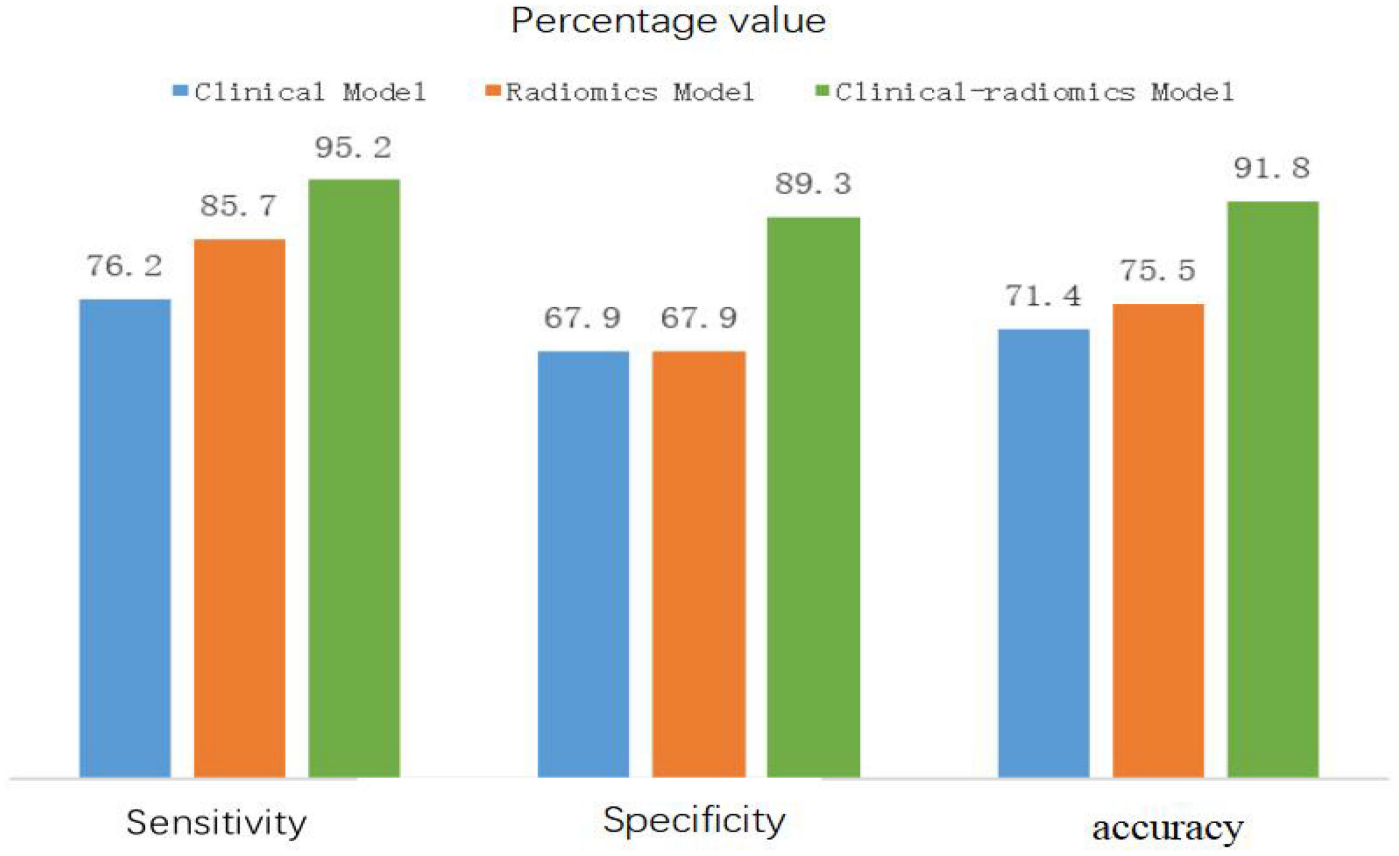
Sensitivity, specificity and accuracy of three models in validation cohort.

**Table 3** shows a comparison of the sensitivity, specificity, accuracy and other diagnostic performance metrics of the clinical model, multimodal ultrasound radiomics model and combined clinical-multimodal radiomics model in the training and validation cohorts. The combined model exhibited the best prediction performance in both the training cohort and the validation cohort, with AUCs of 0.957 (95% CI95% CI: 0.918-0.987) and 0.932 (95% CI95% CI: 0.822-0.984), respectively. In the validation cohort, the discrimination efficiency of the combined prediction model was better than that of the other two models, and the differences were statistically significant (P=0.008; P=0.012). In addition, the sensitivity, specificity and other indicators for predicting CLNM in PTC patients improved after the combination of multimodal radiomic features with clinical and ultrasound features, indicating that, compared with the clinical prediction model, the addition of multimodal ultrasound radiomics can significantly improve the ability of models to predict lymph node metastasis of PTC. A comparison of the ROC curves of the three prediction models in the validation cohort is shown in **Figure 5**, and a comparison of other diagnostic indicators is shown in **Figure 6**.

**Table 3:** Prediction efficacy of different models.

Different models	AUC	95%CI	Sensitivity	Specificity	PPV	NPV	Accuracy
Clinical Model Train cohort	0.841	0.764-0.918	0.673	0.909	0.846	0.789	0.809
Test cohort	0.777	0.636-0.884	0.762	0.679	0.640	0.792	0.714
Radiomics Model Train cohort	0.925	0.865-0.973	0.934	0.849	0.821	0.949	0.887
Test cohort	0.768	0.625-0.876	0.857	0.679	0.667	0.864	0.755
Clinical-radiomics Model Train cohort	0.957	0.918-0.987	0.959	0.864	0.839	0.966	0.904
Test cohort	0.932	0.822-0.984	0.952	0.893	0.870	0.962	0.918

### 3.6 Analysis of the clinical decision curve

**Figure 7:**
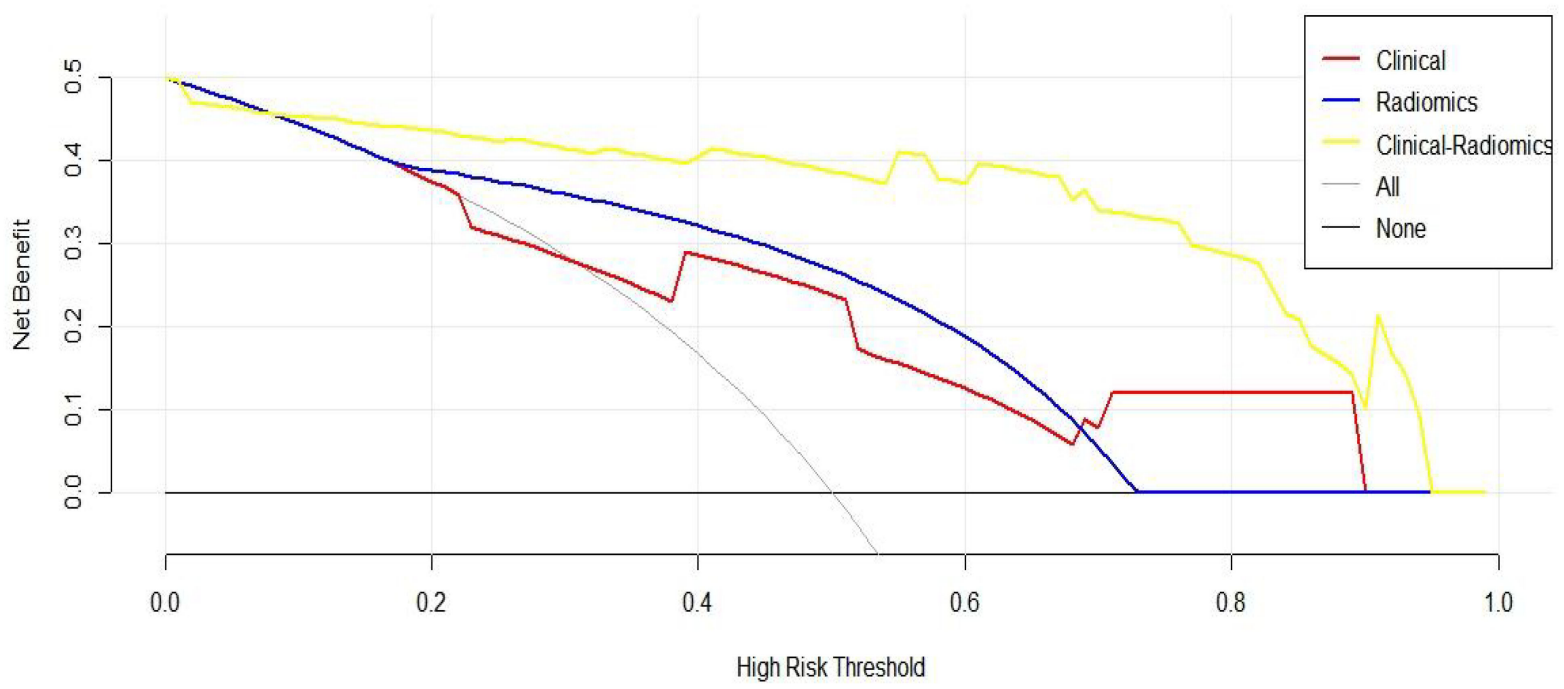
Clinical decision curve of three prediction models (The x-axis represents the onset risk threshold, and the y-axis indicates the net benefit. The black solid line indicates that all PTC patients are assumed to have negative lymph node metastasis and do not receive any treatment, and the gray solid line indicates that all PTC patients are assumed to have positive lymph node metastasis and receive treatment).

To further evaluate the clinical utility of the joint prediction model, a clinical decision curve analysis was performed to quantify the net benefit at different threshold probabilities in the validation cohort (**Figure 7**). The results indicate that the clinical prediction model, ultrasound radiomics model and combined model was more beneficial than all treatments and no treatment for evaluating the status of cervical lymph node metastasis of PTC within all threshold probability ranges. Within a threshold probability range of 0-1.0, the combined prediction model had the greatest net benefit compared with the other models in the assessment of the lymph node metastasis status of PTC patients.

## 4 Discussion

In most cases, PTC progresses slowly and is not fatal, as indicated by a 5-year survival rate of more than 99% for patients who undergo treatment (15). However, there are some patients with PTC who experience lymph node metastasis or thyroid capsular invasion. At present, surgical resection is the main treatment for PTC. For patients suspected of lymph node metastasis, prophylactic dissection of central or lateral lymph nodes is needed, but whether prophylactic lymph node dissection is beneficial for PTC patients is controversial. Dobrinja et al. (16) reported that compared with those who underwent thyroidectomy alone, PTC patients with CN0 who underwent prophylactic cervical lymph node resection had significantly increased risks of temporary recurrent laryngeal nerve injury (p < 0.009) and permanent hypothyroidism (p < 0.016). Therefore, early identification of CLNM in PTC patients can not only help determine the appropriate scope of surgery and reduce the risk of postoperative complications but also lower the risk of recurrence, prevent the need for reoperation, and improve patient prognosis.

Studies have revealed that LNM can be confirmed on the basis of ultrasound findings and clinical features of PTC (17). In this study, tumor size, multifocality and a capsular contact range > 50% were independent risk factors for CLNM, and a clinical prediction model was constructed on the basis of these factors. The AUCs of the models in the training cohort and validation cohort were 0.841 and 0.777, respectively, suggesting that the ultrasound features of PTC may be helpful for the preoperative prediction of CLNM. CHNAG et al. (18) suggested that the abutment/perimeter ratio is an independent risk factor for CLNM. In contrast, in the present study, researchers evaluated lymph node metastasis not only in central lymph nodes but also in lateral cervical lymph nodes and further quantified capsular invasion according to transverse or longitudinal tumor invasion. The results indicate a capsular contact range >50% as an independent predictor of lymph node metastasis, which was consistent with the findings of our previous study (19). However, ultrasound features alone are not sensitive enough to indicate lymph node metastasis. Previous studies (20) have used only ultrasound features to construct models for predicting CLNM, performing univariate and multivariate analyses to identify risk factors for both central lymph node metastasis and lateral lymph node metastasis. Ultimately, they established multivariate logistic regression models with six and seven variables, respectively, to predict central and lateral LNM. In the internal validation cohort, the central lymph node metastasis (LNM) prediction model exhibited an area under the curve (AUC) of 0.702, with a sensitivity of 74.2% and a specificity of 57.6%. Likewise, for the lateral LNM prediction model, the AUC was 0.791, accompanied by a sensitivity of 67.5% and a specificity of 77.1%, as observed within the same validation group. As evidenced, the clinical-multimodal ultrasound radiomics model demonstrates superior performance in predicting CLNM in PTC, particularly in terms of accuracy and AUC, compared to models based solely on conventional ultrasound features. This enhanced performance may be attributed to radiomics’ capability to systematically analyze a multitude of features within ultrasound images, mitigating the potential for subjective interpretation by clinicians. It underscores the potential value of our model in real-world clinical applications, especially in resource-limited settings, where it can assist in rapid and accurate diagnostic decision-making.

In recent decades, radiomics has been applied in research evaluating the status of lymph nodes in patients with malignant tumors. In a previous study, Jiang et al. (21) designed an ultrasound-based radiomics system that combines key clinical risk factors and contrast-enhanced ultrasound radiomic features to predict lymph node metastasis in PTC patients, and the nomogram better predicted the diagnosis of LNM, with AUCs of 0.820 and 0.814 in the training and validation sets, respectively. Liu et al. (22) retrospectively analyzed the ultrasound images of 450 primary tumors in PTC patients. After screening, a prediction model was created and a decision curve analysis was performed on the basis of 50 radiomic features. These results suggest that an ultrasound-based radiomics system is clinically valuable and performs well in the noninvasive evaluation of lymph node metastasis in patients with PTC.

In previous studies, an ultrasound-based radiomics model was constructed on the basis of radiomic features extracted from ultrasound images (23, 24), and few studies have revealed the predictive performance of a multimodal ultrasound-based radiomic system for the evaluation of cervical lymph node metastasis of PTC. Therefore, on the basis of previous studies, this study involved the combination of US, CEUS and SE-US images and the application of radiomics methods to extract high-throughput radiomics features. It is not only a simple multimodal ultrasound-based radiomics prediction model but is also constructed on the basis of statistically significant variables in the univariate analysis of the clinical prediction model in the feature set. After feature dimension reduction and screening, a clinical-multimodal ultrasound radiomics model was constructed to analyze its diagnostic efficiency.

The area under the curve (AUC) of the multimodal ultrasound radiomics model was 0.925 in the training cohort and 0.768 in the validation cohort, with a sensitivity, specificity and accuracy of 85.7%, 67.9% and 75.5%, respectively. In contrast, the AUC of the clinical-multimodal ultrasound radiomics model was 0.957 in the training cohort and 0.932 in the validation cohort, and its sensitivity, specificity and accuracy were as high as 95.2%, 89.3% and 91.8%, respectively, in the validation cohort. Moreover, the diagnostic efficiency of the combined model was not only better (AUC: 0.932 vs. 0.777, P < 0.05) but the sensitivity and specificity of the model were also better than those of the prediction model, which used only clinical factors. These results indicate that if sufficient clinical risk information is provided before surgery, the combined radiomics model based on machine learning will be superior to clinical sonography in assessing the status of lymph node metastasis of PTC, which is consistent with the findings of previous research on the application of AI technology in the evaluation of thyroid tumors (25, 26). Jiang et al. (12) used dual-modality ultrasound images as well as B-US and SE-US images to construct an ultrasound-based radiomics model to evaluate the status of lymph nodes in PTC patients, and the AUC of the final prediction model was 0.851. Compared with the above studies, this study included CEUS images and clinical risk factors in the feature set for the construction of a prediction model that combines the radiomic features extracted from the images of the four modes of ultrasound and clinical risk factors. The model ultimately showed better prediction performance (AUC=0.932 in the validation cohort), which significantly improved the accuracy of preoperative ultrasound in predicting lymph node metastasis of PTC.

A good cancer risk prediction model is a simple but clinically significant mathematical tool designed on the basis of dependent variables and independent variables. Therefore, we plotted a decision curve to further quantitatively assess the true benefit of the combined prediction model. The results indicated that (**Figure 7**) the combined prediction model provided PTC patients with the best net benefit under any threshold probability, which confirmed the feasible application of this model in clinical practice and suggested its relevance as a reliable tool for accurate preoperative diagnosis of lymph node metastasis in patients with PTC.

Some limitations of our study should be acknowledged. First, some bias may inevitably exist and affect our analysis. This is because radiomics models require the mining of several datasets for hidden biological information necessary for the prediction of metastasis. Although we included the images of four modes of ultrasound, the sample size was still insufficient, and our data were not derived from public databases. Prospective multicenter validation using a larger group of patients is needed to acquire high-level evidence for further clinical application. Second, in this study, one frame of ultrasound images with the best performance was selected for each modality, but a single image may not be representative or convincing enough, which may ultimately affect the establishment of radiomics models. In the future, we can obtain more valuable information from more layers of ultrasound images. Third, images were manually segmented for the delineation of the ROI. A research (27) have developed a multimodal deep learning model called DeepThy-Net to predict different CLNM patterns in PTC, the experimental results in two independent test sets showed that the AUC values exceeded 0.87, indicating clinical applicability. In addition, there is a research that has developed an AI-generated content-enhanced computer-aided diagnosis model for thyroid nodules, called ThyGPT (28), which not only automatically generate initial contour suggestions but also continuously optimize results based on user feedback, thereby promoting human-machine collaboration. By training models to recognize specific patterns, the required human resources can be significantly reduced. Although manual segmentation is relatively accurate, it is time-consuming and labor-intensive, and the technology needed for automated segmentation must be further developed.

## Data Availability

The original contributions presented in the study are included in the article/supplementary material. Further inquiries can be directed to the corresponding author.
